# Heavy menstrual bleeding and association with menstruation-specific resources: A multinational cross-sectional study in low- and middle-income countries

**DOI:** 10.1016/j.healthplace.2025.103576

**Published:** 2025-11

**Authors:** Zarmeen Shakil, Bethany A. Caruso, Madeleine Patrick, Thea L. Mink, Tanushree Bhan, Tanvir Ahmed, Jenala Chipungu, Malini Reddy, Chibwe Beatrice Chiwala, Sheela S. Sinharoy

**Affiliations:** a Hubert Department of Global Health, Rollins School of Public Health, Emory University, Atlanta, USA; b Department of Civil Engineering, Bangladesh University of Engineering and Technology, Dhaka, 1000, Bangladesh; c International Training Network - Bangladesh University of Engineering and Technology (ITN-BUET), Dhaka, Bangladesh; d Centre for Infectious Disease Research in Zambia (CIDRZ), Lusaka, Zambia; e Athena Infonomics LLC, India; f Lusaka Water Supply and Sanitation Company (LWSC), Lusaka, Zambia

**Keywords:** Menorrhagia, Menstrual health, Quality of life, Women's health, Sanitation

## Abstract

Heavy menstrual bleeding (HMB), clinically defined as excessive menstrual blood loss which interferes with a woman's physical, emotional, social, and/or material quality of life, is a highly prevalent yet understudied global health problem. This cross-sectional study examines associations between HMB and menstruation-related resources, to understand factors that may contribute to HMB.

We conducted secondary analyses of household survey data collected from women in eight cities across five countries: Meherpur and Saidpur, Bangladesh; Narsapur, Tiruchirappalli, and Warangal, India; Dakar, Senegal; Kampala, Uganda; and Lusaka, Zambia. HMB was assessed using the clinically validated SAMANTA scale, and menstruation-specific resources were measured through validated scales for safety and security, privacy, financial assets, and time. We conducted regression analyses, controlling for demographic covariates and clustering, of associations between HMB and menstruation-specific resources.

Among our analytic sample of 3962 participants, 44.7 % were categorized as experiencing HMB. Results indicated that financial dependence on others for menstruation-related expenses and limited control over time were significantly associated with higher prevalence of HMB. Specifically, each one-point increase in financial dependence was associated with a 9 % higher HMB prevalence (*p* = 0.008), while greater control over time was associated with a 20 % lower prevalence (*p* < 0.001). The use of menstrual materials not specifically designed for menstruation, such as cotton wool or toilet paper, was also significantly associated with higher HMB prevalence.

These findings highlight the critical importance of control over menstruation-specific resources, particularly financial resources and time, as well as improved access to affordable and high-quality menstrual materials, for menstrual health in urban populations.

## Highlights

• 44.7 % of the 3962 survey participants experienced heavy menstrual bleeding (HMB).
• HMB was linked to financial dependence on others for menstruation-related expenses.
• Greater control over time for meeting menstrual needs was linked to lower HMB.
• No significant relationship was found between HMB and privacy or safety/security.
• Use of “other” menstrual materials was associated with higher HMB prevalence.

## 1 Introduction

Heavy menstrual bleeding (HMB) is clinically defined as excessive menstrual blood loss which interferes with a woman's physical, emotional, social, and/or material quality of life (Munro et al., 2018; National Institute for Health Care Excellence (NICE), 2018). This definition was proposed by the UK National Institute for Health Care Excellence (NICE) in 2007 and adopted by the International Federation of Gynecology and Obstetrics in 2018, superseding a previous definition of HMB as menstrual blood loss of 80 mL or more per menstrual period (Munro et al., 2018; National Institute for Health Care Excellence (NICE), 2018). In recommending the new definition, NICE emphasized the inherently subjective nature of HMB and the necessity of responsiveness to “the physical, social and emotional experiences a woman has, rather than … objective measurements defined by a test.” (National Collaborating Centre for Women's and Children's Health (Great Britain), 2007) Evidence suggests that HMB negatively affects women's daily functioning, results in increased absenteeism from school and work, and adds to the existing financial burden women face due to higher healthcare expenses associated with managing adverse menstruation-related symptoms (Ding et al., 2019; Swe et al., 2022; Karlsson et al., 2014).

To date, research on HMB has predominantly concentrated on high-income countries, focusing on underlying biological and clinical factors, the challenges associated with diagnosis, and treatment and management (Davila and Alderman, 2020; Herman et al., 2016; Da Silva Filho et al., 2021; Zia and Rajpurkar, 2016; Vannuccini et al., 2022). The most comprehensive study of HMB to date in low- and middle-income countries (LMICs) measured the prevalence of HMB among women in ten cities across five countries in South Asia and Eastern, Western, and Southern Africa and found that approximately 49 % of women in the study population were experiencing HMB (Sinharoy et al., 2023a). The study observed associations of HMB with adverse health outcomes, including physical symptoms of anemia, worse self-rated physical health, and worse subjective wellbeing. However, the underlying factors influencing the prevalence and experiences of HMB in LMICs remain largely unknown.

The experience of HMB may be influenced by a range of biological, social, environmental, and economic factors. Biologically, HMB may result from structural (e.g., polyps, fibroids, malignancies) and non-structural (e.g., endometrial pathology, ovulatory dysfunction, bleeding disorders) entities (Munro et al., 2018). However, individuals may be classified as experiencing HMB even when their actual volume of menstrual blood loss is within the physiologically normal range if their perceived volume negatively impacts their quality of life (Sinharoy et al., 2023a; Elledge et al., 2018; Schmitt et al., 2018; Hennegan et al., 2019). Indeed, a cross-sectional survey conducted across ten countries (predominantly high-income but also including two upper-middle-income countries, Brazil and China) found that in most cases of HMB (61 %), no structural or haematological cause was identified, suggesting that non-biological causes may be predominant (Lunardi Rocha et al., 2018). Non-biological factors may include social factors such as stigma around menstruation, behavioral norms and expectations related to menstrual management, and knowledge of menstrual health practices, all of which can shape how individuals experience HMB (Hennegan et al., 2019).

The experience of HMB may also be influenced by environmental factors, including physical resource limitations such as inadequate access to sanitation facilities (Hennegan et al., 2019). Approximately 145 million people globally lack access to improved sanitation (JMP, 2025). Importantly, the definition of ‘improved’ sanitation is primarily concerned with separation of human excrement from human contact; it does not consider features like cleanliness, safety or privacy of the sanitation facility (Caruso et al., 2024; JMP. Sanitation, 2024). If these essential features were considered, estimates of those lacking access to an adequate sanitation facility would likely be higher. Specifically, across menstrual health research, cleanliness, safety, and privacy have been identified as essential features of locations within which individuals who menstruate can maintain their menstrual, physical, and mental health (Hennegan et al., 2019; Barrington et al., 2021). These locations should be safely accessible and free from threats of violence or stigma to enable effective menstrual management (Swe et al., 2022; Hennegan et al., 2019; Rossouw and Ross, 2021; Girod et al., 2017). Yet, one study found that more than 70 % of individuals across eight LMICs lack access to safe, clean, and private menstrual hygiene management spaces (Rossouw and Ross, 2021). Other critical resources within clean, safe, and private facilities include access to soap and water for personal washing and bins for material disposal (Schmitt et al., 2018; Hennegan et al., 2023). Beyond the facility, access to an enclosed bathing space and a water source on the household compound are also critical (Caruso et al., 2020). Inadequate sanitation facilities, both at home and away, can hinder women and girls from performing essential menstrual care tasks, compromising their menstrual health and quality of life, and potentially contributing to HMB (Hennegan et al., 2021).

Beyond the household, built environments that provide facilities such as latrines with menstrual products are essential for menstrual health management. An audit of public toilets in six cities (spanning low-to high-income countries) revealed major access disparities (Nguyen et al., 2025). Transit hubs had the highest concentration of public toilets, followed by business districts, and residential areas had the fewest. Business districts, which often benefit from dedicated maintenance, were more likely to have menstruation-friendly facilities. However, fewer than 3 % of public facilities offered menstrual products (free or for purchase), and 64 % had disposal bins (Nguyen et al., 2025). Those living in urban areas may have better access to sanitation facilities but still face challenges accessing adequate facilities (Rossouw and Ross, 2021). For example, women in urban India, despite better access to latrines than women in rural and tribal areas, report higher fears of sexual assault when using these facilities (Hulland et al., 2015). These findings underscore that even in urban settings where sanitation access is more readily available, the quality, safety, and adequacy of facilities often do not meet menstruators’ needs.

Accessible, affordable, and high-quality menstrual materials are another important resource. In a pooled sample of 47 Multiple Indicator Cluster Surveys conducted between 2017 and 2023 across 44 countries, comprising data from 673,380 women and girls, 95 % of respondents reported use of any menstrual materials (defined as pads, tampons, or cloth) (Starr et al., 2025). However, use of menstrual materials varies widely within and across LMICs, based on urban/rural settings and demographic characteristics such as wealth (Rossouw and Ross, 2021). Women in urban areas are significantly more likely to use disposable pads compared to their rural counterparts, who often rely on different materials of varying quality (Rossouw and Ross, 2021). For instance in Madagascar, individuals in urban areas were three times more likely to use pads, while those in rural areas were five times more likely to report not using any menstrual materials, highlighting barriers to access within countries. Girls and women in LMICs and in rural areas are more likely to use reusable materials, such as cloth, and few have enough menstrual materials to change as often as needed (JMP, 2025). For example, in Ethiopia, although 82 % of women report using menstrual materials, only 46 % indicate having easy access to them (JMP, 2025). In many low-resource settings, cloth remains a common material, despite not being designed for menstruation; this can result in leaks and staining, which may reduce quality of life and contribute to HMB (Chinyama et al., 2019).

These differences in access to sanitation facilities and menstrual materials are further shaped by financial resources. Research across eight LMICs have found that individuals with greater financial resources were more likely to have access to lockable, private, and safe sanitation facilities (Rossouw and Ross, 2021; Jaafar et al., 2023). For instance, in Lao People's Democratic Republic, 97 % of the wealthiest women use menstrual materials compared to 47 % of the poorest (JMP, 2023). Financial resources were also identified by a systematic literature review as being an important factor in the type of menstrual material used, with many studies across LMICs describing a “lack of funds to purchase menstrual items … and lack of affordable cloth or commercial menstrual products,” and that the top choice of menstrual material is often unaffordable or considered a luxury (Hennegan et al., 2019). A lack of financial resources may compel women to use menstrual products for extended periods, increasing the risk of infections, and negatively impacting quality of life and HMB experiences (Jaafar et al., 2023).

Control over time also may be a relevant factor in HMB experiences, as it is necessary for accessing sanitation facilities, changing materials, bathing, and managing other menstruation-related needs. Studies with both schoolgirls and working women have identified control over time as an essential menstruation-specific resource (McMahon et al., 2011; Borg et al., 2023). These populations were found to face constraints that prevented timely changing of menstrual materials or bathing, frequently leading to the suppression of urination and postponement of menstrual health needs (Borg et al., 2023). Societal pressures to conceal menstruation at home, school, and in the workplace, expectations to bathe extensively to maintain cleanliness, and domestic water collection and other household responsibilities, can further exacerbate these challenges (Barrington et al., 2021; Hennegan et al., 2020; Caruso et al., 2022; Graham et al., 2017). These pressures coincide with the need to maintain discretion, creating additional strain (Hennegan et al., 2020; Caruso et al., 2022). Therefore, control over time is essential for individuals to meet menstrual health needs (Caruso et al., 2017). Lack of control over time may contribute to HMB experiences as individuals may have to delay, avoid, or suppress necessary care.

Research on non-biological contributors to HMB is limited, specifically regarding menstruation-specific resources that may contribute to experiences of HMB in LMICs settings. To address this gap, we empirically examined the association between menstruation-specific resources and HMB. Our research question was: *What is the association between access to menstruation-specific resources (safety and security; privacy; financial assets; and time) and the experience of HMB in LMICs?* By quantifying this relationship, we aimed to identify specific modifiable factors that are associated with HMB, which could inform future research and policy to enhance resources and support better health outcomes for women experiencing HMB in LMICs.

## 2 Materials and methods

### 2.1 Study design, participants, and procedures

This is a secondary analysis of cross-sectional data collected as part of the Measuring Urban Sanitation and Empowerment (MUSE) project, which aimed to develop and validate quantitative survey instruments to measure women's empowerment in relation to sanitation in urban areas of LMICs. Data were collected in eight cities: Meherpur and Saidpur, Bangladesh; Narsapur, Tiruchirappalli, and Warangal, India; Dakar, Senegal; Kampala, Uganda; and Lusaka, Zambia. These cities were selected purposively in collaboration with the funder, the Bill & Melinda Gates Foundation, because of their involvement in the Citywide Inclusive Sanitation (CWIS) program.

Within each city, we worked with local partners to purposively select neighborhoods for survey implementation based on partner priorities (e.g., focusing on formal or informal settlements). Neighborhoods were defined administrative units such as zones in Kampala and wards in Indian cities. The total number of neighborhoods sampled for the MUSE survey ranged from 9 in Lusaka to 43 in Saidpur. Local field teams consisting of trained female enumerators who were hired, trained, and supervised by local partners then randomly selected households within each neighborhood using a random walk technique and sought participation of an adult woman within each selected household. Inclusion criteria for the MUSE survey were: being a woman aged 18 or older who spoke the primary local language in each location; being mentally competent, without any disabilities that would preclude participation in the survey; and being a full-time resident of the household. Additional details of study design, original survey, and procedures are described elsewhere (Sinharoy et al., 2022, 2023b, 2024).

Survey instruments were translated and independently back-translated into local languages, programmed on tablets with KoBo Toolbox in Bangladesh and Uganda and Ona software in all other sites, and administered in person by the field teams. The survey included modules on demographics; water, sanitation, and hygiene access and behaviors; scales to measure sanitation-related empowerment, and other measures for validation purposes, which have been described in detail elsewhere (Sinharoy et al., 2023b, 2023c). The survey also included a question asking whether the respondent had experienced a menstrual period in the past 12 months. If the answer to that question was ‘yes’, the respondent was asked additional questions about menstrual practices and experiences, as described in further detail below.

The survey for the MUSE project was conducted with a total of 5707 women from August 2021 to June 2022. The analytic sample for the secondary analysis presented here was restricted to women who reported having experienced a menstrual period in the past 12 months and had complete data for all variables included in the models. We removed observations with missing data (*n* = 26), along with those who did not report having a menstrual period in the last 12 months (*n* = 1746) and those who did not respond to the HMB question(s) (*n* = 16). Upon additional review, we identified three coding errors and after consulting with the field team, two individuals were excluded, and one was recoded. This resulted in a final analytic sample of 3962 women.

### 2.2 Measures

#### 2.2.1 Outcome: Heavy menstrual bleeding (HMB)

The primary outcome, HMB, was measured using the SAMANTA scale, which was developed and validated in a clinical population in Spain and then further validated among non-clinical populations in the LMIC populations described here (Sinharoy et al., 2023a; Calaf et al., 2020). The SAMANTA scale includes six questions that focus on perceived volume and abundance of menstrual blood loss; length in days of the menstrual period; experiences of spotting or avoiding activities due to the need to frequently change menstrual materials; and subjective experiences of being bothered or feeling worried due to menstruation. The six items of the SAMANTA scale are shown in Appendix 1. The response options for all six questions are “yes/no”. Scoring was conducted in alignment with Calaf et al. (2020), which specifies that affirmative responses to two specific questions (“Do you experience menstrual bleeding for more than 7 days per month?” and “In general, does menstruation bother you due to its abundance?“) should each receive three points, while affirmative responses to all other items receive one point (Calaf et al., 2020). These values are summed, resulting in a potential range of values for the HMB score from 0 to 10. Individuals with scores of at least 3 are then classified as experiencing HMB while individuals with scores below 3 are categorized as not experiencing HMB.

#### 2.2.2 Primary exposures: Menstruation-specific resources

We examined women's perceptions of four menstruation-specific resources: safety and security; privacy; financial assets; and time. These four resources were selected based on prior literature suggesting their importance for influencing menstruation-related quality of life and therefore contributing to HMB, as described above. Three of the resources (safety and security; privacy; and time) were measured using scales and financial assets was measured using a single item. The three scales, as well as the single item for financial assets, had all been developed and validated as part of the MUSE project (Sinharoy et al., 2022, 2023b, 2023c, 2024). Operational definitions of the four menstruation-specific resources, along with items and corresponding response options, are shown in Table 1, and details of validation are available elsewhere (Sinharoy et al., 2023b, 2023c, 2024).

**Table 1:** Operational definitions, example items, and response options for measurement of menstruation-specific resources.

Resource	Operational definition	All items	Response options
Safety and security	Women's freedom from acts or threats of violence when accessing locations to change menstrual materials	1. During my last menstrual period, I felt safe in the place where I typically went to change my menstrual materials/manage my menstruation when I was at home. 2. During my last menstrual period, I felt safe in the place where I typically went to change my menstrual materials/manage my menstruation when I was away from home. 3. During my last menstrual period, the location I used to change my menstrual materials/manage my menstruation during the day was safe at night.	Responses ranging from 1 = never to 4 = always
Privacy	Women's ability to maintain desired levels of privacy when managing menstruation and accessing locations to manage menstruation	1. During my last menstrual period, I was worried that someone would see me changing my menstrual materials/managing my menstruation while using a sanitation location. 2. During my last menstrual period, I was worried that someone would see me carrying menstrual materials on my way to a sanitation location. 3. During my last menstrual period, I had to use locations to change my menstrual materials/manage my menstruation that were not private enough for me. 4. During my last menstrual period, I had difficulty finding a private place to change my menstrual materials/manage menstruation. 5. During my last menstrual period, I worried that someone will see my menstrual blood in the sanitation location that I use to change my menstrual material. 6. During my last menstrual period, I worried that someone would see me washing my menstrual materials in a sanitation location.	Responses ranging from 1 = never to 4 = always
Financial assets	Women's control over economic resources and long-term stocks of value such as land, for the purposes of meeting individual menstruation needs	1. I depend on someone else to pay for menstruation-related expenses.	Response ranging from 1 = strongly disagree to 4 = strongly agree
Time	Women's control over their time spent on menstruation-related tasks and activities	1. I would like to have more time to meet my menstruation-related needs. 2. I often have to wake up earlier than I want to access a location to manage my menstruation. 3. I often miss out on activities I would like to do because of time spent taking care of my menstruation-related needs.	Responses ranging from 1 = strongly disagree to 4 = strongly agree

Menstruation-specific safety and security, privacy, time, and financial assets were assessed through items using Likert scale responses (Table 1). Safety and security was measured using three items with response options of 1 = never, 2 = sometimes, 3 = often, and 4 = always, with higher scores indicating higher perceived menstruation-related safety and security. Privacy was assessed using six items with the same four response options (ranging from 1 = never to 4 = always), which were reverse coded such that higher values corresponded to better perceived menstruation-related privacy. Time was measured using three items with the same response options, ranging from 1 = strongly disagree to 4 = strongly agree, which were reverse coded such that higher scores indicated better perceived control over time to meet menstruation-related needs. For each construct, scores were created by calculating a mean score of the relevant item responses, resulting in continuous scale scores used in the current analysis. The financial assets item was assessed with response options of 1 = strongly disagree, 2 = disagree, 3 = agree, 4 = strongly agree, where a higher score indicated greater economic dependence on others for fulfilling menstruation-related needs (i.e., a higher score indicates less control over financial assets).

#### 2.2.3 Covariates

The analysis included several covariates related to demographic characteristics and menstrual practices, which were selected from the original survey a priori based on the literature (Sinharoy et al., 2022, 2023b, 2024). Specifically, demographic characteristics used as covariates were age in years, included as both a linear and a quadratic term to account for potential non-linear associations with HMB; highest level of completed schooling, a categorical variable coded as less than primary, primary, and beyond primary education; and city, representing the eight cities in the analysis. Both age and education have been associated in the literature with menstrual management, with no formal education or primary education linked to poorer menstrual management, and younger age groups facing more challenges across menstrual health compared to older age groups (Ghosh and Bose, 2021).

The covariates related to menstrual practices included in our analyses were: type of menstrual material used most often in the past three cycles; usual disposal method of menstrual materials; and type of sanitation facility. Menstrual materials used most often were categorized as reusable products (cloth, reusable pads, menstrual cups, and absorbent underwear/period panties), disposable products (tampons and single use/disposable pads), and other materials (toilet paper, cotton wool, natural materials such as leaves or grass, mattress or foam, underwear alone, no materials used, and other). Use of reusable products was coded as 1, disposable products as 2, and ‘other materials’ as 3. These categories are derived from existing literature and encapsulate the distinct practical concerns associated with each type of menstrual material. Disposable products require accessible waste disposal systems, while reusable products necessitate facilities for proper washing and drying (Fourcassier et al., 2022). The “other materials” category was kept separate as these materials may be poorly suited or less effective as menstrual absorbents (Hennegan et al., 2019).

The usual disposal methods for menstrual materials were categorized into disposal in a trash bin (either inside or outside the house) and other disposal methods. The ‘other disposal methods’ category included disposal options such as flushing down the toilet or pit latrine, discarding in an open drain, leaving outside in the open, burning, burying outside, and instances where there was no disposal. Disposal in a trash bin was coded as 1 and other disposal methods were coded as 0. Disposal into trash bins was categorized separately, as it reflects the presence of an enabling environment where trash bins are readily available for menstrual waste disposal. The higher frequency of this practice in certain LMIC cities compared to others suggests variations in the availability and quality of infrastructure for waste disposal (Kattimani et al., 2024). In contrast, limited access to trash bins in many LMIC cities highlights inequities in sanitation infrastructure and poses significant health and environmental risks (Elledge et al., 2018; Kattimani et al., 2024; Winter et al., 2022; Sommer et al., 2013).

Type of sanitation facility had three categories: shared sanitation with others outside of household, unshared latrine (i.e., a latrine that is used by one household exclusively), or private stall in a shared latrine block. These were coded as 1, 2, and 3 respectively. Importantly, access to an unshared latrine or a private stall in a shared latrine block does not necessarily imply privacy, which is measured separately. Shared sanitation facilities are common in LMICs but, in some contexts, have been associated with higher risks of adverse health outcomes, such as diarrheal diseases, when compared to individual household latrines (Heijnen et al., 2014; Sprouse et al., 2024). However, the evidence supporting an increased risk of these adverse outcomes remains weak and underexplored thus, for this analysis, we included individual household latrines along with two levels of sharing, as shared sanitation may serve as a viable alternative for larger populations without access to individual household latrines as recommended by prior literature (Heijnen et al., 2014). This categorization captures differences in both individual household latrines and types of shared sanitation.

### 2.3 Statistical analysis

Descriptive statistics were calculated to examine the total and city-level distribution of population characteristics. Three multivariable Poisson regression models were then developed after assessing multicollinearity among the variables using the variance inflation factor (VIF). All individual VIFs were below 1.3, and the mean VIF was 1.16, indicating no evidence of multicollinearity among the exposure variables. Poisson regression is appropriate for use with cross-sectional data in which a binary outcome is not rare, as is the case with our outcome of HMB (Barros and Hirakata, 2003).

**Fig. 1:**
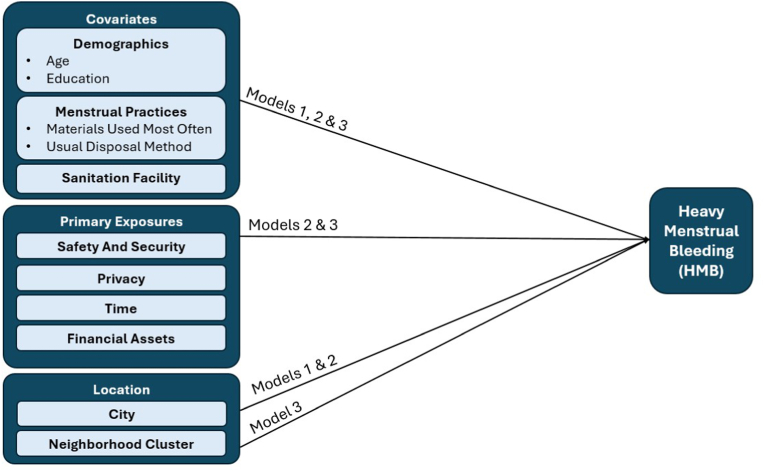
Heavy menstrual bleeding and its association with menstruation-specific resources.

To assess relationships between HMB and our selected covariates, we first built a model (Model 1) with HMB as the dependent variable and demographic characteristics (age, education, city), menstrual practices (menstrual materials used most often, usual disposal method), and type of sanitation facility as independent variables. For the second model (Model 2), to examine the associations between HMB and menstruation-specific resources while adjusting for covariates, we included all variables from the first model and added the four independent variables for menstruation-specific safety and security, privacy, financial assets, and time. Finally, to account for potential non-independence of observations that could be due to socio-economic status or community-level infrastructure for water, sanitation, hygiene, and menstrual health, we built a third model (Model 3) that included all variables from the first and second models but dropped the variable for city and instead controlled for clustering at the neighborhood level. Neighborhoods within each city were designated as strata to account for potential clustering effects using the ‘svy’ command to consider the complex survey design. Fig. 1 illustrates the three analytic models.

All analyses were conducted in Stata SE 17.0 (StataCorp, 2025).

## 3 Ethics

Study protocols were reviewed and approved by ethics review committees in each site: Emory University, USA (IRB00110271), Makerere University in Kampala, Uganda (MAK-SHSREC Ref No. 2019-038), ERES Converge in Lusaka, Zambia (2020-Oct-013), National Health Research Authority in Zambia (NHRA00005/May 13, 2021), Comité National d’Ethique pour la Recherche en Santé in Dakar, Senegal (SEN21/85), International Institute of Health Management Research in New Delhi, India (IRB/2020–2021/001), and International Training Network-Bangladesh University of Engineering and Technology in Dhaka, Bangladesh (1.003/2022/02). Participants in all cities received compensation (either cash or in-kind) per local policies and ethical requirements. All women provided informed consent before participation in the study. The funder was involved in discussions related to the purposive selection of cities for data collection. The funder had no role in data collection, data analysis, data interpretation, or writing of the manuscript.

## 4 Results

### 4.1 Descriptive results

#### 4.1.1 Demographic characteristics

The analytic sample consisted of 3962 participants (Table 2). Demographic characteristics for these participants are shown by city in Table 2. The mean age of participants was 31.8 years, ranging from 18 to 56 years old. Most of the participants were educated beyond primary school (*n* = 2,586, 71.4 %), and only 7.6 % of the participants had less than primary education.

**Table 2:** Demographic characteristics of participants across 8 Sub-Saharan and South Asian cities (n = 3962).

Cities (in alphabetical order)																		
	Dakar		Kampala		Lusaka		Meherpur		Narsapur		Saidpur		Tiruchirappalli		Warangal		Total	
	(n = 329)		(n = 414)		(n = 439)		(n = 552)		(n = 463)		(n = 599)		(n = 609)		(n = 557)		(N = 3962)	
	n	Mean	n	Mean	n	Mean	n	Mean	n	Mean	n	Mean	n	Mean	n	Mean	n	Mean
**Age**	329	31.71	414	30.16	439	30.74	552	32.43	463	31.3	599	30.18	609	32.68	557	34.38	3962	31.79
Range	18–50		18–50		18–56		18–52		18–49		18–50		19–50		19–55		18–56	
**Schooling**	**n**	**%**	**n**	**%**	**n**	**%**	**n**	**%**	**n**	**%**	**n**	**%**	**n**	**%**	**n**	**%**	**n**	**%**
Less than Primary	10	4.2	40	9.83	16	3.77	44	8.29	54	13.88	50	8.85	20	3.42	41	8.45	275	7.59
Primary education	87	36.55	96	23.59	125	29.48	70	13.18	114	29.31	89	15.75	86	14.73	95	19.59	762	21.03
Beyond primary	141	59.24	271	66.58	283	66.75	417	78.53	221	56.81	426	75.4	478	81.85	349	71.96	2586	71.38
**Type of Sanitation**	**329**		**414**		**439**		**552**		**463**		**597**		**609**		**555**		**3958**	
Shared with others	125	37.99	306	73.91	314	71.53	98	17.75	23	4.97	50	8.38	105	17.24	37	6.67	1058	26.73
Not shared	202	61.4	88	21.5	125	28.47	452	81.88	440	95.03	547	91.62	471	77.34	518	93.33	2844	71.85
Private stall	2	0.61	19	4.59	0	0	2	0.36	0	0	0	0	33	5.42	0	0	56	1.41
**Type of Disposal**	**329**		**411**		**436**		**552**		**463**		**599**		**608**		**557**		**3955**	
In trash bin	5	1.52	224	45.5	35	8.03	214	38.77	406	87.69	337	56.26	361	59.38	431	77.38	2332	58.96
Other	324	98.48	187	54.5	401	91.97	338	61.23	57	12.31	262	43.74	247	40.62	126	22.62	1623	41.04
**Menstrual Materials Most Often Used**	**328**		**413**		**432**		**551**		**463**		**598**		**609**		**557**		**3951**	
Reusable products	9	2.74	56	13.56	32	7.41	238	43.19	130	28.08	272	45.48	99	16.26	114	20.47	950	24.04
Disposable products	291	88.72	347	84.02	366	84.72	226	41.02	333	71.92	233	38.96	506	83.09	440	78.99	2742	69.4
Other	28	8.54	10	2.42	34	7.87	87	15.79	0	0	93	15.55	4	0.66	3	0.54	259	6.56
**Experiences of HMB**	**329**		**414**		**439**		**552**		**463**		**599**		**609**		**557**		**3962**	
Reported HMB	126	38.3	158	38.16	226	51.48	260	47.1	208	44.92	283	47.25	256	42.04	254	45.6	1771	44.7

In this sample, 44.7 % of respondents were categorized as experiencing HMB (*n* = 1771). In South Asia, the prevalence of HMB was relatively consistent across cities, being lowest in Tiruchirappalli (42.0 %) and highest in Saidpur (47.3 %). More variation was seen in African cities, where HMB prevalence ranged from 38.2 % in Kampala (and 38.3 % in Dakar) to 51.6 % in Lusaka.

#### 4.1.2 Menstrual practices

Among the menstrual materials used most often, disposable products were reported most frequently (*n* = 2,742, *M* = 69.4). In African cities, the use of disposable products ranged from 84.0 % in Kampala to 94.7 % in Lusaka. In South Asian cities, it ranged from 38.9 % in Saidpur to 83.1 % in Tiruchirappalli. Reusable products were used by 24.0 % (*n* = 950) of participants overall, with the lowest usage reported in Dakar (2.7 %) and the highest in Saidpur (45.5 %). The “other” category was selected by 6.6 % (*n* = 259) of participants overall, with the highest usage observed in Meherpur (15.8 %) and Saidpur (15.6 %).

More than half of participants (*n* = 2,332, *M* = 59.0) reported usually disposing of their menstrual materials in a trash bin, though this percentage ranged from 1.5 % in Dakar to 87.7 % in Narsapur. All South Asian cities with the exception of Meherpur had a higher prevalence of usual disposal of menstrual materials in a trash bin compared to African cities (over 50 %). In African cities, self-reported usual disposal in a trash bin was 1.5 % in Dakar, 45.5 % in Kampala, and 7.8 % in Lusaka. Self-reported frequency of “other” disposal methods was 41.0 % (*n* = 1623) and was highest in Dakar (98.5 %) and lowest in Narsapur (12.3 %).

A majority of the sample did not share their sanitation facilities with others outside their household (*n* = 2,844, 71.9 %). However, this percentage varied widely across cities. In Kampala, only 21.5 % of women reported not sharing their sanitation facilities, while in Narsapur, 95.0 % did not share their facilities. In African cities, more women shared their sanitation facilities with others outside their household (*n* = 745, 63.0 %) than in South Asian cities (*n* = 313, 11.3 %).

#### 4.1.3 Primary exposure: Menstruation-specific resources

All four menstruation-specific resources; safety and security; privacy; time, and financial assets, were measured on a 1 to 4 scale, with higher scores indicating greater access, except for financial assets, where higher scores indicate greater economic dependence. Privacy had the highest mean score (*M* = 3.8, SD = 0.4), with a notably skewed distribution (skewness = −2.6), indicating a strong perception of privacy among most respondents. Safety and security also had a high mean score (*M* = 3.6, SD = 0.7) and negative skew (−1.68), reflecting a similar concentration of higher scores. In contrast, time had a mean score of 2.9 (SD = 0.6) with minimal skewness (−0.15), indicating a more balanced distribution across scores. Financial assets had the lowest mean (*M* = 2.5, SD = 0.8) and minimal skewness (0.04), indicating limited financial control among many respondents.

### 4.2 Regression results

In Model 1, HMB was not significantly associated with age, education, or type of sanitation facility. However, HMB was significantly associated with type of menstrual material used most often, with a positive association between HMB and the use of ‘other’ menstrual materials (prevalence ratio (PR = 0.25, *p* = 0.015) (Table 3).

**Table 3:** Association between HMB, demographic, and menstruation-resources-domain across 8 Sub-Saharan and South Asian cities (n = 3962).

Prevalence ratio (PR), standard error, confidence interval, p-value												
	Demographic Model (n = 3604)				Demographics and Resources Model (n = 3.597)				Clustered by Neighborhood in Each City: Demographics and Resources Model (n = 3597)			
	Model 1				Model 2				Model 3			
	PR	SE	CI	P value	PR	SE	CI	P value	PR	SE	CI	P value
**Age**	−0.04	0.25	(-0.08, 0.12)	0.145	−0.03	0.02	(−0.08, 0.02)	0.201	−0.03	0.02	(−0.07, −0.00)	0.062
**Age^2**	0.00	0.00	(-0.00, 0.00)	0.254	0.00	0.00	(-0.00, 0.01)	0.292	0.00	0.00	(-0.00, 0.00)	0.136
**Schooling**												
Primary school (ref)												
Less than Primary	0.01	0.11	(-0.21, 0.22)	0.992	−0.01	0.11	(−0.22, 0.21)	0.948	−0.01	0.08	(−0.17. 0.15)	0.906
Beyond primary	0.07	0.10	(-0.13, 0.26)	0.509	0.07	0.10	(−0.13, 0.26)	0.514	0.07	0.67	(−0.06, 0.20)	0.298
**Type of Sanitation**												
Shared with others (ref)												
Not shared	−0.01	0.07	(-0.16, 0.13)	0.839	0.02	0.73	(−0.12, 0.16)	0.770	0.04	0.05	(−0.06, 0.13)	0.459
Private stall	−0.33	0.27	(-0.86, 0.19)	0.211	−0.30	0.27	(−0.82, 0.22)	0.267	−0.35	0.20	(−0.74, 0.05)	0.084
**Type of Disposal**	−0.02	0.06	(-0.14, 0.10)	0.757	−0.01	0.06	(−0.13, 0.11)	0.840	−0.06	0.04	(−0.13, 0.01)	0.115
**Menstrual Materials Most Often Used**												
Reusable products (ref)												
Disposable products	−0.01	0.07	(−0.14, 0.13)	0.890	0.01	0.07	(−0.13, 0.14)	0.921	−0.02	0.05	(−0.12, 0.07)	0.637
Other	0.25	0.10	(0.05, 0.45)	0.015	0.26	0.10	(0.06, 0.46)	0.011	0.28	0.06	(0.16, 0.41)	<0.001∗
**City**												
Kampala (ref)												
Tiruchirappalli	0.13	0.11	(−0.09, 0.35)	0.235	0.02	0.11	(−0.20, 0.24)	0.862				
Saidpur	0.20	0.12	(−0.03, 0.43)	0.092	0.18	0.12	(−0.05, 0.42)	0.131				
Lusaka	0.25	0.11	(0.04, 0.47)	0.022	0.15	0.11	(−0.07, 0.38)	0.173				
Dakar	0.00	0.14	(−0.27, 0.28)	0.977	−0.08	0.14	(−0.36, 0.20)	0.545				
Narsapur	0.24	0.12	(−0.00, 0.48)	0.054	0.05	0.13	(−0.20, 0.31)	0.703				
Warangal	0.21	0.12	(−0.02, 0.44)	0.073	0.04	0.12	(−0.20, 0.28)	0.758				
Meherpur	0.18	0.12	(−0.04, 0.41)	0.115	0.12	0.12	(−0.11, 0.35)	0.309				
**Safety and security**					−0.07	0.42	(−0.16, 0.01)	0.076	−0.07	0.04	(-0.15, 0.00)	0.06
**Privacy**					−0.01	0.07	(−0.13, 0.14)	0.941	0.02	0.06	(−0.10. 0.13)	0.762
**Financial assets**					0.09	0.03	(0.02, 0.15)	0.008	0.09	0.03	(0.03, 0.14)	0.001
**Time**					−0.20	0.05	(-0.29, −0.11)	<0.001∗	−0.19	0.03	(−0.25, −0.12)	<0.001∗

In Model 2, in which the four menstruation-specific resources (safety and security, privacy, financial assets, and time) were added to the model, financial assets and time were significantly associated with HMB. Financial assets were positively associated with HMB (PR = 0.09, *p* = 0.008), indicating that individuals with greater financial dependence on others for menstruation-related expenses (higher scores) were more likely to experience HMB. Specifically, a one-point increase in the financial assets score was associated with a 9 % higher likelihood of HMB compared to those with less financial dependence for menstruation-related expenses. Scores for time were negatively associated with HMB (PR = −0.20, *p* < 0.001), indicating that women with greater control over time for meeting menstruation-related needs were less likely to experience HMB compared to those with lower control over their time to meet their menstruation-related needs. Each one-point increase in the time score (i.e., the mean score on the time scale) was associated with a 20 % decrease in prevalence of HMB. No associations were observed between HMB and either safety and security (PR = −0.07, *p* = 0.076) or privacy (PR = −0.01, *p* = 0.941) (Table 3).

Model 3 remained consistent with Model 2, after adjusting for neighborhood clustering. Specifically, associations of HMB with scores for financial assets (PR = 0.09, *p* = 0.001) and time (PR = −0.19, *p* < 0.001) remained consistent, and there was a significant and positive relationship between HMB and use of ‘other’ menstrual products (PR = 0.28, *p* < 0.001) (Table 3). The result for ‘other’ menstrual products indicates that the prevalence of HMB among those who most often use ‘other’ menstrual materials is 28 % higher than those who primarily use reusable products (reference group). Similar to Model 2, safety and security and privacy remained non-significant in Model 3.

## 5 Discussion

This study examined associations between HMB and menstruation-specific resources, to better understand how non-biological factors may be related to the subjective experience of HMB. We observed that HMB was significantly associated with financial assets for menstruation-related expenses and control over time to meet menstruation-related needs. HMB was also significantly associated with the use of ‘other’ menstrual materials (i.e., those that are not designed as menstrual absorbents, such as cotton wool or toilet paper). To our knowledge, ours is the first study to empirically examine the association between menstruation-specific resources and HMB and, as such, contributes important new knowledge to our understanding of factors that may contribute to HMB among women in LMICs. More broadly, our study highlights the importance of comprehensively investigating the full range of factors that may contribute to the experience of HMB across LMIC settings, beyond biological causes.

Our finding of a significant association between menstruation-related financial assets and HMB extends prior literature on period poverty, described as insufficient access to menstrual products and sanitation facilities (Rossouw and Ross, 2021; Jaafar et al., 2023; Rohatgi and Dash, 2023). Specifically, in our results, women with greater financial dependence on others to meet menstruation-related needs were more likely to experience HMB. This association may be due to economic constraints and gendered social norms leading women to be dependent on others to pay for their menstruation-related expenses, hindering their access to menstrual health resources and adequate menstrual care and impairing their menstruation-related quality of life. Previous research has found that financial insecurity and poverty limit women and girls' ability to purchase preferred menstrual products, contributing to significant challenges in managing menstruation (Hennegan et al., 2019; McMahon et al., 2011). Financial insecurity may also affect access to sanitation facilities that provide privacy and safety. For example, using nationally representative data from eight LMICs on menstrual health practices among women aged 15–49 across Sub-Saharan Africa, South, and South-East Asia, Rossouw and Ross (2021) found that women and girls from less wealthy households were less likely to have access to clean, private, safe, and lockable spaces, as well as soap, water, and menstrual materials, compared to those from wealthier households (Rossouw and Ross, 2021). Global inequality has deepened due to the COVID-19 pandemic and the ongoing effects of climate change, which are likely to worsen period poverty and further limit women's access to adequate menstrual materials and resources (Michel et al., 2023; Harrison and Tyson, 2023; Alugnoa et al., 2022).

Our observation that greater control over time for meeting menstruation needs was associated with lower HMB scores also aligns with prior research, which has mainly focused on school-going girls and working women. School girls face time constraints in managing menstrual health needs, such as bathing privately without stigma (McMahon et al., 2011). Among working women, a study in Uganda indicated that 61 % of women reported needing to delay urination, and 35 % were unable to change their menstrual materials when they wanted (Borg et al., 2023). Additionally, the pressure for working women to appear ‘responsible’ by being discreet and maintaining cleanliness reinforces gender-inequitable judgments surrounding menstruation and increases the time burden of extensive body washing (Hennegan et al., 2020). Our study expands on these previous findings by generating additional evidence from a broader study population of how limitations on women's control over time to meet menstruation-related needs may be linked to their quality of life and, therefore, HMB.

The lack of significant associations of HMB with privacy and safety and security was surprising, considering that previous research across LMICs consistently highlights the critical role of privacy, safety, and security in menstrual health across populations of women and girls (Hennegan et al., 2019; Rossouw and Ross, 2021). For example, research across eight LMICs found a significant proportion of women were unable to lock their menstrual management facilities, and women in the Democratic Republic of Congo and Ethiopia reported concerns about the lack of privacy and safety associated with these facilities (Rossouw and Ross, 2021). Other research has found privacy, safety and security to be important concerns for school-going girls accessing menstruation facilities in various LMICs (Swe et al., 2022; Girod et al., 2017; Sommer et al., 2015). In Kenya, girls reported harassment and assault following the onset of menstruation, with boys attempting to peek into bathroom stalls or ridiculing them for menstrual stains (Girod et al., 2017). In Myanmar, girls feared teasing by boys upon their menstruation status being known, particularly while using latrines or changing menstrual products in sanitation facilities (Swe et al., 2022). Private, lockable, and safe spaces for menstrual management are likely to be important for menstrual-related quality of life, though they did not predict HMB specifically in our study populations.

Among our covariates, we found a strong and statistically significant association between the use of “other” products that are not intended primarily for use as menstrual materials, such as cotton wool or toilet paper, and HMB. Access to menstrual materials such as disposable pads remains a challenge in many low-resource contexts due to factors such as cost, distance to vendors, limited market access, reliance on others to purchase pads, and shame around pad disposal (Caruso et al., 2017). As a result, using “other” materials may be more affordable and accessible for women, particularly those whose access to preferred menstrual materials is constrained by economic barriers and the availability of functioning markets (Schmitt et al., 2017). Women financially dependent on others may be more likely to rely on “other” materials, aligning with our findings that limited economic resources are associated with higher HMB, suggesting hindered access to adequate and preferred menstrual materials. Our results suggest a need to not only measure the sufficiency of menstrual materials, as recommended by the WHO/UNICEF Joint Monitoring Programme (JMP), but also to consider individuals' access to their preferred materials (Priority, 2024). This expanded approach would allow for a comprehensive assessment of whether individuals have enough materials, whether those materials align with their preferences, and whether they can access their preferred menstrual materials, which may ultimately impact their menstrual health (Smith et al., 2020).

### 5.1 Strengths and limitations

An important strength of this study is the large and diverse sample of women from eight cities in five countries across regions of Africa and South Asia, which increases the generalizability of our findings. At the same time, because the study design is cross-sectional, only associations with HMB can be identified, not causal relationships. Both our primary exposures and outcome were self-reported measures, which may be subject to social desirability and recall bias. For example, given the stigma surrounding menstruation in some contexts, participants may have underreported experiences of HMB or had difficulty accurately recalling past menstrual experiences (Shapley et al., 2012). Furthermore, self-reported measures used to assess menstruation-specific resources may not fully capture the complexity of access, such as the influence of cultural practices.

Since this was a secondary analysis, we were unable to control for biological or clinical causes of HMB, which may have impacted our results. However, the use of a validated scale for HMB - previously validated and applied among both clinical and non-clinical populations - as well as for menstruation-specific resources, validated on non-clinical populations, reduces the risk of bias and increases confidence that we are accurately measuring the intended constructs. Additionally, we were unable to include a measure of socioeconomic status or a wealth index, which can be critical factors influencing menstrual health and access to other key resources (Rossouw and Ross, 2021). Finally, our sample included only women aged 18 and above, potentially overlooking gender-diverse people who menstruate and adolescent girls who may be disproportionally affected by HMB (Borzutzky and Jaffray, 2020). We recommend further research with these populations, particularly in rural and low-resource settings, that includes standardized measures of household wealth or socioeconomic status to better understand how economic inequality shapes the experience of HMB.

## 6 Implications for research, programs, and policy

Based on our study results, future research and programs should explore underlying pathways of HMB and the socioecological factors that can be targeted to alleviate its burden while enhancing women's menstrual health in LMICs. Research questions could include more comprehensively examining the relationship between menstrual health resources, safety considerations, sanitation facilities, and HMB as well as the impact of initiatives such as reduced taxation on menstrual products or improved access to affordable, high-quality menstrual products in resource-limited settings. With respect to programs, our results highlight the need for sanitation programs in LMICs, along with public health programs more broadly, to contribute to increasing access to physical and economic resources, including material and infrastructural resources, for meeting menstruation-related needs. Specifically, addressing HMB requires a comprehensive socioecological approach that incorporates both physical resources and systemic factors such as education, healthcare access, and economic empowerment. Programs should adopt a multi-sectoral strategy that engages communities, researchers, and policymakers, leveraging public-private partnerships and government funding. Raising awareness and driving systemic change could ensure greater investment in menstrual health, ultimately providing better support for women with HMB and enhancing their overall well-being.

Our results provide additional support for existing recommendations that policy initiatives should address menstruation-related resource constraints, particularly for menstrual materials and adequate sanitation facilities for washing, changing, and disposal, to improve menstrual health (Barrington et al., 2021). Specifically, these efforts should prioritize increasing access to affordable, high-quality menstrual materials, while reducing both economic and physical barriers that hinder women's ability to purchase necessary products and access and use sanitation facilities in LMICs. As noted above, research has indicated that women and girls, particularly in poor urban and rural communities of LMICs, are less likely to access and use disposable pads or their other preferred menstrual materials due to financial constraints and lack of affordability (Chandra-Mouli and Patel, 2017). Increased taxation on menstrual products exacerbates gender inequities (Rossouw and Ross, 2021). Policy reform is needed to remove taxes on menstrual products, making these products more affordable and improving access. However, the effectiveness of these policies needs to be further examined to ensure they create tangible improvements in women's economic ability to manage their menstrual health with more autonomy and control over their time.

## 7 Conclusion

This study highlights the association between HMB and menstruation-specific resources necessary for effective menstrual health management in LMICs. We identified three factors that were significantly associated with HMB: financial dependence on others for menstruation-related expenses, control over time to meet menstruation-related needs, and the use of ‘other’ menstrual materials not designed for menstruation. These findings underscore the importance of targeted interventions to alleviate the economic and time-related burdens on women associated with menstrual health and hygiene and to improve access to high-quality menstrual materials. Future research should investigate the underlying socioecological pathways of HMB to better empower women experiencing menstrual challenges, ensuring that sanitation interventions address both physical and economic barriers. A holistic approach that integrates infrastructure development while addressing systemic issues like economic empowerment is essential for improving menstrual health outcomes and the well-being of women with HMB in LMICs.

## Funding sources

This work was supported by the 10.13039/100000865Bill & Melinda Gates Foundation [Grant Numbers OPP1191625 and INV-028835; to 10.13039/100019281SSS and BAC] and the 10.13039/100006939Emory Specialized Center of Research Excellence on Sex Differences (10.13039/100006939Emory 10.13039/501100025022SCORE) [10.13039/100000002National Institutes of Health (10.13039/100000002NIH) award number U54AG062334; to 10.13039/100019281SSS].

## CRediT authorship contribution statement

**Zarmeen Shakil:** Conceptualization, Formal analysis, Methodology, Visualization, Writing – original draft, Writing – review & editing. **Bethany A. Caruso:** Conceptualization, Funding acquisition, Investigation, Project administration, Supervision, Writing – review & editing. **Madeleine Patrick:** Conceptualization, Data curation, Funding acquisition, Investigation, Project administration, Supervision, Writing – review & editing. **Thea L. Mink:** Conceptualization, Data curation, Writing – review & editing. **Tanushree Bhan:** Conceptualization, Data curation, Writing – review & editing. **Tanvir Ahmed:** Funding acquisition, Project administration, Supervision, Writing – review & editing. **Jenala Chipungu:** Investigation, Project administration, Supervision, Writing – review & editing. **Malini Reddy:** Writing – review & editing. **Chibwe Beatrice Chiwala:** Writing – review & editing. **Sheela S. Sinharoy:** Conceptualization, Data curation, Funding acquisition, Investigation, Methodology, Project administration, Supervision, Writing – original draft, Writing – review & editing.

## Declaration of competing interest

Authors have no conflicts of interest to disclose.

## Data Availability

Data will be made available on request.
