# Supplementary material for: Heavy menstrual bleeding and association with menstruation-specific resources: A multinational cross-sectional study in low- and middle-income countries
Source: Health Place. 2025 Nov;96:103576. doi: 10.1016/j.healthplace.2025.103576 (PMC12660180; doi:10.1016/j.healthplace.2025.103576)
Supplement: Multimedia component 1 [file mmc1.docx]

| **Appendix 1: SAMANTA Scale Items** | |
| --- | --- |
| **Items** | **Questions** |
| 1 | Do you experience menstrual bleeding during more than 7 days per month? |
| 2 | Do you experience 3 or more days of heavier menstrual bleeding during your menstrual period? |
| 3 | In general, does menstruation bother you due to its abundance? |
| 4 | During any of these heavier menstrual bleeding days do you spot your clothes at night; or would you spot them if you did not use double protection/did not change your clothes during the night? |
| 5 | During these heavier menstrual bleeding days, are you worried about staining the chair, sofa, etc.? |
| 6 | In general, during these heavier menstrual bleeding days, do you avoid, as far as possible, some activities, trips, or leisure time plans because you frequently need to change your menstrual materials? |
